# Review of ERCP Techniques in Roux-en-Y Gastric Bypass Patients: Highlight on the Novel EUS-Directed Transgastric ERCP (EGDE) Technique

**DOI:** 10.1007/s11894-021-00808-3

**Published:** 2021-07-01

**Authors:** Harshit S. Khara, Swetha Parvataneni, Steven Park, Jihye Choi, Truptesh H. Kothari, Shivangi T. Kothari

**Affiliations:** 1 Department of Gastroenterology, Geisinger Medical Center, Danville, PA 17822 USA; 2 Department of Medicine, Geisinger Medical Center, Danville, PA 17822 USA; 3 Department of Medicine, University of Rochester Medical Center, Rochester, NY 14642 USA; 4 College of Art and Design, Rochester Institute of Technology, Rochester, NY 14623 USA; 5 Department of Gastroenterology, University of Rochester Medical Center, Rochester, NY 14642 USA

**Keywords:** Roux-en-Y gastric bypass (RYGB), Endoscopic retrograde Cholangiopancreatography (ERCP), Endoscopic ultrasound directed transGastric ERCP (EDGE), Device assisted ERCP, Laparoscope-assisted ERCP (LA-ERCP), Gastric access temporary for endoscopy (GATE)

## Abstract

**Purpose of Review:**

Hepatobiliary complications are common in Roux-en-Y gastric bypass (RYGB) patients. Despite development of multiple surgical and endoscopic access techniques over the years, ERCP using standard duodenoscope remains challenging in these patients due to the altered anatomy.

**Recent Findings:**

Limited success with enteroscope-assisted and laparoscope-assisted ERCP led to the evolution of the novel EUS-directed transgastric ERCP (EDGE) procedure, with variations of this technique termed as Gastric Access Temporary for Endoscopy (GATE), EUS-guided TransGastric ERCP (EUS-TG-ERCP), EUS-guided GastroGastrostomy-assisted ERCP (EUS-GG-ERCP), and EUS-directed transgastric intervention (EDGI). EDGE has high technical (100%) and clinical success rates (60–100%), lower adverse event rate (1.5–7.6%), and up to 20% access stent migration rate; without any significant weight changes. EDGE has significantly shorter procedure time (73vs184min), post-procedural hospital stays (0.8vs2.65 days) and is more cost effective compared to other modalities.

**Summary:**

EDGE technique addresses the challenges of RYGB anatomy as a minimally invasive, clinically successful, fully endoscopic, and cost-effective option. We present a literature review of the EDGE technique from its inception to current, in addition to reviewing other access techniques, their advantages, disadvantages and outcomes.

## Introduction

Over the recent years, obesity has emerged as a pandemic in the US and worldwide, contributing to about 400,000 deaths attributable to poor diet and physical inactivity [1]. Although diet and lifestyle modifications are the initial approaches to obesity treatment, their modest outcomes have led to an increased interest in bariatric surgery [2, 3]. Multiple bariatric procedures such as gastric banding, sleeve gastrectomy, and Roux-en-Y-Gastric Bypass (RYGB) have emerged, of which, RYGB has superseded other bariatric procedures by 70%- 80% [4, 5].

**Fig. 1:**
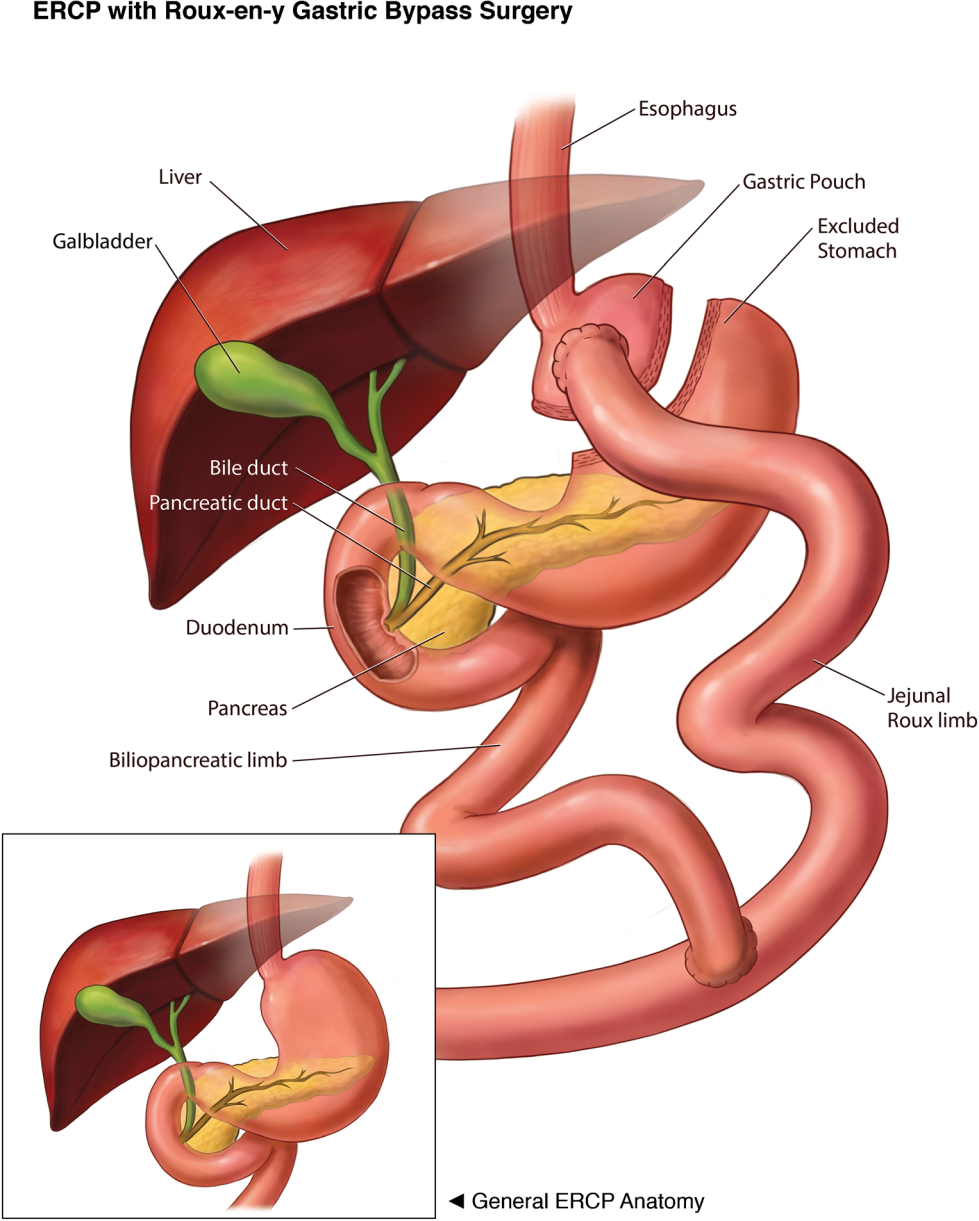
Roux-en-Y Gastric Bypass Anatomy compared to normal ERCP anatomy (inset)

About 29%–36% of post bariatric patients develop gallstones, and 13% develop gallbladder sludge within 6 months to 18 months after surgery [6, 7]. Traditional endoscopic retrograde cholangiopancreatography (ERCP) using a standard duodenoscope to treat pancreaticobiliary disorders is challenging in post RYGB patients because of the altered anatomy (Fig. 1) and the technical difficulty of maneuvering the duodenoscope down the Roux limb to the jejuno-jejunal anastomosis and then up the biliopancreatic limb (short limb 80–100 cm or long limb 100–150 cm) to reach and gain access to the papilla [8, 9].

In this article, we discuss the various endoscopic and surgical techniques that have been developed over the years to address this challenge, with a focus on the efficacy, safety, and comparative outcomes of the newly developed Endoscopic ultrasound-directed transgastric ERCP (EDGE) procedure over other techniques.

## Techniques

### Colonoscope and Enteroscope-Assisted ERCP

In 1988, Gostout and Bender first reported the use of a pediatric colonoscope in 3 patients to reach the papilla in Roux-en-Y anatomy [10]. Later, Elton et al. from 1994 to 1997 (*n* = 18) described their experience using a pediatric colonoscope and enteroscope for diagnostic and therapeutic intervention in long limb bypass patients. In this study, if the procedure with pediatric colonoscope was not successful on first attempt then an enteroscope was used to reattempt the ERCP. With both the endoscopes, they reported an overall success rate of access to papilla or bilio-enteric and pancreatico-enteric anastomoses as 84%, cannulation rate of 94%, and performance of ERCP in 86%. Although success rates were higher, the disadvantages included the lack of side viewing orientation and an elevator with the scope as well as smaller channel size that precluded the use of large diameter stents and accessories [11].

### Duodenoscope

In 1997 Hintze et al. studied the efficacy of conventional duodenoscope and reported a success rate of only 33% in reaching the papilla in RYGB patients, and 67% in patients with Billroth II anastomoses [12].

### Combined Duodenoscope and Colonoscope

Later in 2002, a prospective study reported a 67% ERCP success rate after multiple attempts with the use of a forward-viewing colonoscope and the duodenoscope in long limb RYGB patients with intact papilla. Even though success was achieved on repeat attempts, the number of failed initial attempts even in experienced hands highlighted the need to develop better techniques for this procedure [13].

## Advanced Endoscopy Techniques

The development of advanced endoscopic techniques is broadly classified into two categories: device-assisted enteroscopy and alternative access point creation for use of duodenoscope.

### Device Assisted Enteroscopy (DAE)

Enteroscopes were designed and widely used for the diagnosis and treatment of small bowel diseases. Recently, balloon tip overtube or rotational overtube-assisted procedures such as Double Balloon Enteroscopy (DBE), Single Balloon Enteroscopy (SBE) and Spiral Enteroscopy (SE) have been utilized to perform ERCP in RYGB patients [14].

### Double Balloon Enteroscopy and Single Balloon Enteroscopy

**Fig. 2:**
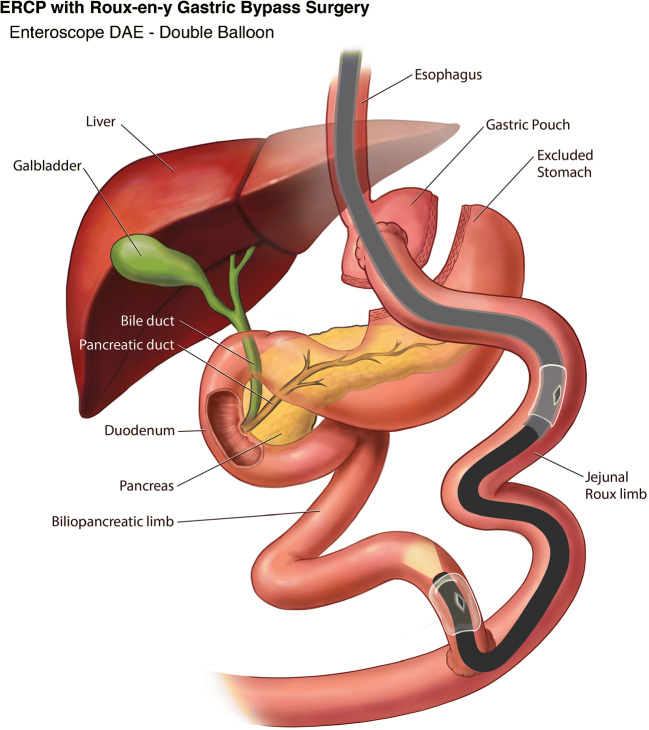
Device-assisted ERCP in Roux-en-Y Gastric Bypass using Double Balloon Enteroscope

**Fig. 3:**
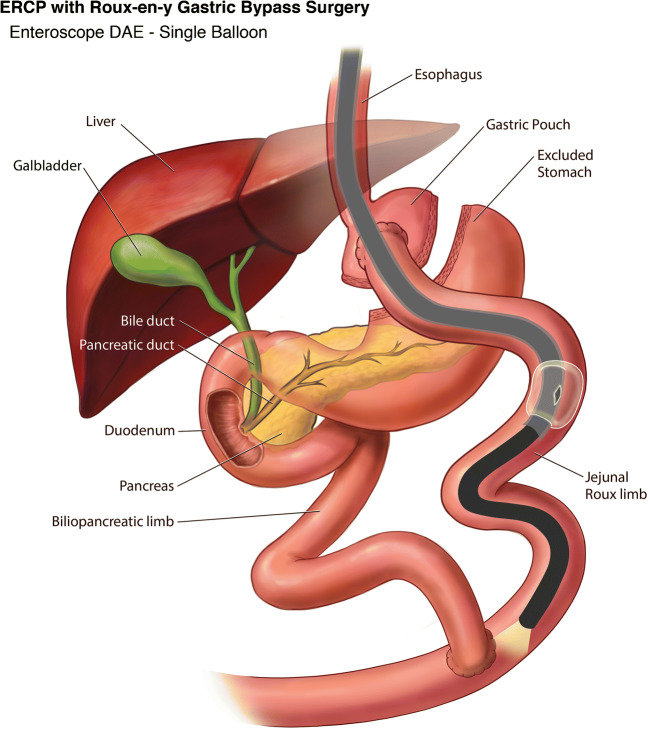
Device-assisted ERCP in Roux-en-Y Gastric Bypass using Single Balloon Enteroscope

DBE was first described in 2001 by Yamamoto et al. as a means of deep exploration of the small bowel [15]. Five years later it was used to perform ERCP in RYGB patients [16]. There is a long and short length DBE scope with lengths of 200 cm and 155 cm respectively, and a working channel of 3.2 mm. The shorter length of the latter scope allows for the use of standard ERCP devices (Fig. 2). Subsequently, the feasibility of performing ERCP in RYGB patients using the single balloon tip overtube was first reported in 2008 [17]. The SBE length is similar to the long DBE scope at 200 cm but with a working channel of 2.8 mm (Fig. 3). In RYGB patients, a systematic review showed that DBE was able to reach the papilla or the anastomosis in 89%, cannulation was successful in 93% with a therapeutic success rate of 82%. Whereas with SBE, papilla or anastomosis was reached in 82%, cannulation was successful in 86% of cases with an overall therapeutic success rate of 68% [18]. Although DBE and SBE demonstrated higher success rate when compared to standard endoscopes, the success rates were more attributed to patients with short Roux limb with bilioenteric anastomosis and intact papilla (80%), compared to 58% with long Roux limb with intact papilla (*p* = 0.040) [19].

### Spiral Enteroscopy

**Fig. 4:**
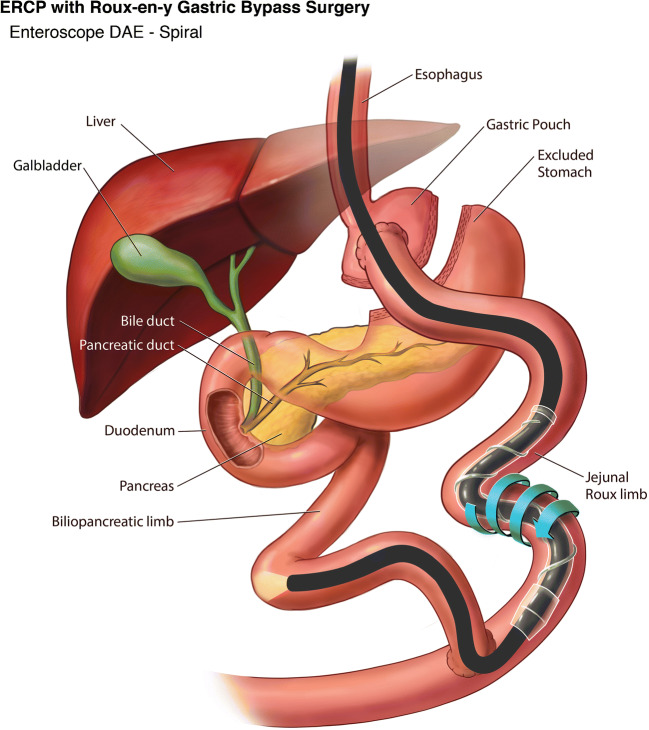
Device-assisted ERCP in Roux-en-Y Gastric Bypass using Spiral Enteroscope

SE was introduced as an alternative to balloon assisted enteroscopy by Akerman and Cantero for the management of small bowel disorders [20]. Two studies so far have described the use of SE to perform ERCP in RYGB patients (Fig. 4). In both the studies, SE was able to reach the papilla in 76.2% to 86% of patients. Once the papilla was reached, cannulation and therapeutic intervention was successful in 92.3% to 100% of patients [21, 22].

#### DAE-ERCP Comparative Studies

Although the reported data with SE alone has shown higher efficacy rates, a large multicenter comparative study of all the three techniques such as DBE, SBE and SE in RYGB reported ERCP technical success rates of 74%, 69% and 72% and clinical success rates of 63%, 60% and 65%, respectively [23••].

The reasons for the limited success rates with forward viewing enteroscopes were the 1) inability to maneuver the endoscope to reach the native papilla due to the long length of the Roux limbs, internal hernias, and/or adhesions leading to sharp angulations; 2) forward viewing nature of the scope makes cannulation of the ampulla difficult due to the caudal approach; 3) lack of an elevator; 4) the long durations of the device assisted procedures; and 5) limited compatible accessories to fit the length and diameter of the scope channel. Even though short overtubes were introduced to overcome some of these limitations, the small working channel remains a challenge for large diameter stent insertion and use of standard biliary accessories. Also, the success rate is dependent on the available expertise at select tertiary care centers and thus difficult to generalize for the community practices.

### Alternative Access

To achieve higher efficacy and success rates, a second technique called alternative access ERCP, which includes Laparoscope-assisted ERCP (LA-ERCP), Percutaneous Assisted Trans prosthetic Endoscopic Therapy (PATENT), and EDGE procedure, was developed to provide the ability to use a standard duodenoscope and thereby the available standard ERCP accessories.

### Laparoscope-Assisted ERCP

**Fig. 5:**
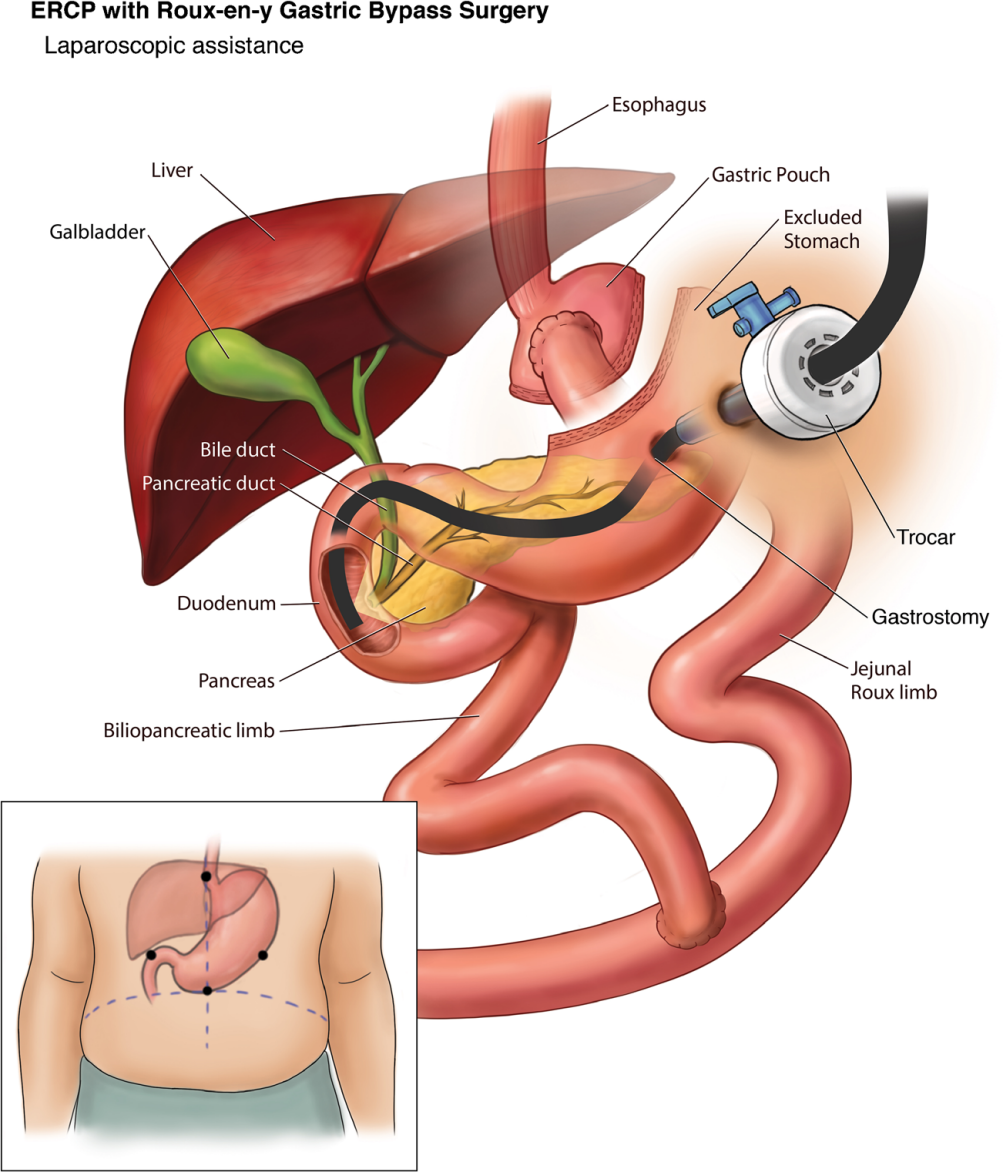
Laparoscope-assisted ERCP in Roux-en-Y Gastric Bypass

LA-ERCP was first described in 2002 [24]. This procedure entails a laparoscope-assisted surgical port placement into the excluded stomach, followed by percutaneous passage of the duodenoscope via the lap port into the duodenum. This facilitates the use of standard accessories via the side viewing duodenoscope (Fig. 5). A systematic review of 509 cases from 26 studies described the feasibility, safety and outcomes of LA-ERCP in patients with RYGB. The study reported 100% successful gastric access and 98.5% successful ductal cannulation [25]. A large multicenter evaluation of 579 patients reported a median procedure time for LA-ERCP to be 152 mins, with median length of hospital stay of 2 days [26•]. In addition to the ERCP success rates, laparoscopic examination facilitates the diagnosis and treatment of adhesions and internal hernias which is a potential morbid complication seen with Roux-en-Y reconstruction [27].

#### LA-ERCP Comparative Studies

LA-ERCP, with more than 95% technical success rates, has surpassed the DAE techniques which has 60–70% technical success rates in the treatment of pancreaticobiliary diseases in RYGB patients [25]. Desai et al. showed that LA-ERCP has a higher success rate (100%) as compared to SE (57%) (*p* = 0.005) [28]. However, the complication rate was 11% higher with LA-ERCP when compared to DAE, and 80% of these complications were related to the gastrostomy site [25, 29, 30].

Although LA-ERCP reported higher technical and clinical success rates when compared with DAE, its limitations include the need for higher technical expertise, more resource utilization due to operating room use as opposed to endoscopy suite, need for sterilizing the scope, and coordination of the surgical and endoscopist schedules [27, 31, 32]. The expertise of the surgeon working alongside the endoscopist is also very important. In patients with high BMI, multiple adhesions, prior surgeries, and the ability to access to the bypassed stomach can be technically challenging and time consuming. The endoscopist also must be experienced in navigating the duodenoscope through the trocar and positioning it in the duodenum through the bypassed scope.

To mitigate the complications associated with laparoscopic creation of a gastrostomy tract, some institutions have reported ERCP via gastrostomy tract created by interventional radiologists, but this can only be performed in a non-emergent setting [33].

However, the above studies highlighted the need of a complete endoscopic approach which overcomes the above disadvantages, leading to the evolution of another alternative access ERCP procedure such as PATENT and EDGE techniques.

### Percutaneous Assisted Trans Prosthetic Endoscopic Therapy (PATENT)

#### DAE-Guided PATENT

PATENT technique was first described by Baron et al. in 2012 [34]. This technique was designed with an intent to develop a complete endoscopic approach by placing percutaneous gastrostomy (PEG) tube and subsequent performance of ERCP via the PEG in RYGB patients. This technique was demonstrated in 9 pigs and 1 human case in 2012 [35••]. The technical success rate of PEG and stent placement was 100%, but cholangiography was successful only in three animals. Stent migration and peristomal infection were the two adverse events reported in the animals.

**Fig. 6:**
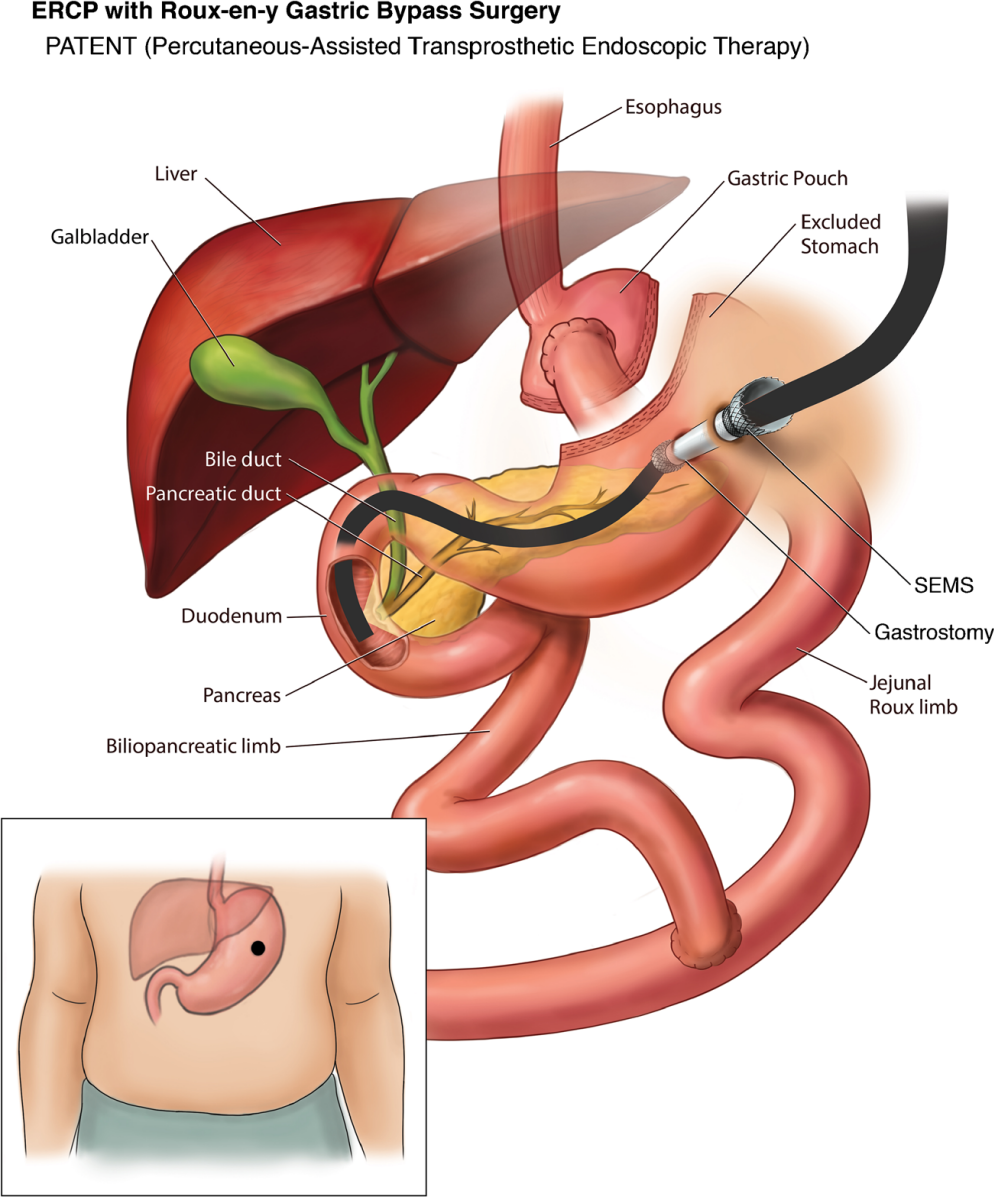
Percutaneous-assisted Transprosthetic Endoscopic Therapy (PATENT) ERCP in Roux-en-Y Gastric Bypass

Later, in 2013 a retrospective case series by the same group demonstrated the use of PATENT technique in 5 patients [36]. All patients underwent transoral DAE-assisted (DBE n  =  4; SBE n  =  1) gastrostomy creation in the excluded stomach with the use of three T-tags in a triangular configuration around the intended PEG site to secure apposition of the gastric and abdominal walls. After sequential dilation of the PEG site, a fully covered self-expanding esophageal metal stent (FCSEMS) was deployed within the gastrostomy tract. The SEMS was then maximally expanded and a standard duodenoscope was advanced through the percutaneous SEMS and the distal stomach to perform antegrade ERCP (Fig. 6). After the ERCP was completed, a 26-Fr balloon bumper PEG tube was placed at the gastrostomy site, which was subsequently removed no sooner than 4 weeks after the procedure to allow for tract maturation. The median procedure time of intubation of the enteroscope to PEG placement was reported as 97 min (IQR 76–186). Sphincterotomy induced perforation in one patient was the only reported complication in the study.

#### EUS-Guided PATENT

PATENT technique was further modified by Law et al. in 2015 when endoscopic ultrasound (EUS) guidance was used instead of DBE for the placement of PEG tube access [37]. The approach of using EUS for placement of PEG in RYGB patients was previously described in 2011 by Attam et al., wherein it was primarily done for feeding tube placement but they also used it to perform ERCP via the PEG site in one patient [38].

Although the PATENT technique is an endoscopic approach, it still involves creation of a gastrostomy site with significant complications of the PEG site access and longer procedure times. Thus, there was still a need for the development of a completely endoscopic and minimally invasive technique without the need for percutaneous access or gastrostomy creation.

### EUS-Directed Transgastric ERCP (EDGE)

#### Initial Two-Stage EDGE Technique

EDGE procedure was first described by Kedia et al. in 2013 as a two-stage procedure. In the first stage, a 16 Fr gastrostomy tube was placed percutaneously into the excluded stomach using EUS guidance to identify and distend the excluded stomach via the gastric pouch. In the second stage, a FCSEMS was exchanged at the gastrostomy site, and an antegrade ERCP was performed using a side viewing duodenoscope that passed through the stent to reach the area of the papilla, very similar to the previously described EUS-guided PATENT technique. This study included 6 patients who underwent a two-stage EDGE procedure. Initial access was successful in 5 of 6 patients (83%). In one patient, first attempt was unsuccessful due to loss of wire access to the bypassed stomach. A mean wait time between the two stages was 5.8 days (± 2.2 days). Antegrade ERCP (second stage) was successfully performed in all 6 patients (100%). Though the procedure had higher success rate, it cannot be performed in emergent situations such as cholangitis because of the two-stage approach. In addition, PEG site infection was noted in 2 of 6 patients (33%), thus revealing the same limitations of the PATENT procedure [39].

#### Novel Single-Stage EDGE Technique

With the advent of the lumen apposing metal stent (LAMS), Kedia et al. in 2014 described a case of a single stage EDGE procedure by creating an EUS-directed gastro-gastric fistula using the LAMS in RYGB patient to perform antegrade ERCP. This was the first report of an entirely endoscopic internal EDGE procedure that could be performed by a single team in a minimally invasive fashion at a single session [40•]. A follow up single-center case series by Kedia et al. on this internal EDGE technique showed successful EUS-directed gastrogastric (*n* = 4) and EUS-directed jejunogastric (*n* = 1) access in all 5 patients (technical success 100%) with the 15 mm diameter LAMS. ERCP was successfully performed during the index procedure in 3 of 5 (60%) patients, but in 2 of 5 (40%) patients ERCP was postponed due to difficulty in passing the duodenoscope through the LAMS on the initial procedure. No adverse events such as bleeding, perforation, peritonitis or pancreatitis were reported. Stent dislodgement was seen in 3 of 5 cases, 2 of which required a second LAMS and in 1 patient the LAMS was readjusted back into position. Removal of the LAMS and fistula closure with endoscopic suturing was confirmed in 2 of 5 patients, and LAMS was left in place in 3 patients for continued biliary access. No weight gain was reported in these patients on follow up. The mean time of procedure was 68 min [41•].

#### EDGE Safety & Efficacy

A multicenter study by Ngamruengphong et al. (*n* = 13) evaluated the safety and efficacy of EDGE but coined the name as EUS-guided transgastric (EUS-TG) ERCP. Technical success rate for placement of 15 mm LAMS and clinical success rate of ERCP through LAMS was 100%. The median wait time to perform ERCP after the LAMS placement was 11 days. Stent dislodgement was seen in 33% in whom therapeutic duodenoscope was used, but none with the slim duodenoscope. Similarly, an interim analysis by Tyberg et al. (*n* = 16) reported technical success of 100% and clinical success of 91%. Unlike the prior study they did not comment on the wait period between the LAMS placement and ERCP, but stent dislodgement was seen in 19% in whom a FCSEMS was used for replacement of LAMS. In both the studies, interventions such as over the scope clip (OTSC), endoscopic suturing, and argon plasma coagulation (APC) were used for closure of the fistulous tract, whereas some patients were left to heal by secondary intention. On follow up there was a mean weight change of −2.85 kg to - 3.6 kg [42, 43].

Some studies have assessed the fistula status in addition to technical and clinical success rates of the procedure. A retrospective analysis of 19 patients from 2018 by James et al. aimed to assess the fistula closure rate after the LAMS removal and describe the associated signs and symptoms of persistent fistula and methods of closure. Technical success of LAMS placement was 100%. ERCP was performed during the index procedure in 4 patients while the remaining 15 patients had ERCP (*n* = 11) and EUS (*n* = 4) after a mean wait period of 48 days from LAMS placement. Interestingly this is the first study wherein they used LAMS to perform EUS guided diagnostic biopsy in RYGB patients. Similar to the study by Tyberg et al., this study also managed the stent dislodgement with FCSEMS. LAMS removal was performed at a mean of 182 days ± 158 days. APC was routinely performed on 12 patients to close the fistulous tract, except in 7 others who required repeat pancreaticobiliary access via the fistula tract. On mean follow up at 281 days, upper GI series was obtained in 11 patients to assess the fistula status. One of 11 (9%) had persistent fistula and gained about 5.6 kg, successfully closed with APC followed by OTSC placement at the jejunogastric access site, leading to subsequent weight loss of 2.8 kg. Mean cohort weight change was +1.7 kg [44•].

A multicenter study presented by Runge et al. at DDW 2019 assessed the success, long-term complications and implications following the EDGE technique. Total of 166 patients were included in the study from 12 centers. Technical success was 98%, gastrogastric access was 52% and jejunogastric in 48% of cases. LAMS was anchored in 21% (35/166) of patients (with suturing in 25, plastic double pigtail stents in 7, hemoclips in 2, and OTSC in 1). EDGE was performed in a single session in 51% and in two sessions in 49% of cases. On mean follow up time of 47 days, LAMS was removed in all patients; and fistula closure was performed in 73% of patients; whereas 27% were left undisturbed following LAMS removal. Upper GI series was obtained in 51% (85/166) of patients, of which 10 patients (12%) had persistent gastrogastric fistula (GGF) and endoscopic closure was performed in 7 of 10 patients with a mean of 1.2 attempts. Intraprocedural and delayed complications were reported in 17% (28/166) of patients [45].

#### EDGE as GATE

A single center case series from 2019 by Wang et al. proposed a new management algorithm for EDGE cases, also coined as Gastric Access Temporary for Endoscopy (GATE). The technical success for LAMS placement as well as clinical success rate was 100% in 10 patients, 3 gastrogastric and 7 jejunogastric, for 9 ERCP cases, and 1 case of EUS followed by endoscopic submucosal dissection (ESD) of a duodenal mass. In 7 of 9 patients ERCP was done during the index procedure and in the remaining 2 patients ERCP was performed in 2–3 weeks, after fistula tract maturation. In 3 of 7 patients in whom ERCP was done at the time of index procedure, LAMS was exchanged with a double pigtail plastic stent immediately after the procedure as their gastric remnant access site was transgastric. In the remaining 4 patients, 3 had stent exchanged later as their access site was transjejunal and 1 had LAMS left in place with an intent of an additional follow up ERCP. Three patients were lost to follow up, and of the remaining 7 patients, all had LAMS exchanged for double pigtail plastic stents. Of these 7, 5 cases had the plastic stent spontaneously expelled and the tract had closed, and the remaining 2 had plastic stent removed manually. All 7 of 7 cases (100%) had confirmed access tract closure. Two patients (20%) had adverse events such as bleeding and stent dislodgment both of whom were transjejunal access [46•].

#### EDGE as EDGI

Prior studies demonstrated the use of EDGE in performing ERCP in RYGB anatomy, but a multicenter study by Kraft et al. from 2019 coined a new term called EUS-directed transgastric intervention (EDGI), discussing the use of this technique in evaluation of various luminal and extraluminal conditions such as pancreatic mass, inflammatory pancreatic fluid collection, suspected cholangiocarcinoma, idiopathic recurrent pancreatitis, common bile duct dilation, abnormal liver function tests, duodenal mass, duodenal stricture, duodenal ulcer perforation, abnormal gastric imaging on CT scan, etc. Extraluminal interventions included EUS-guided drainage pancreatic fluid collection, EUS-guided fine needle aspiration of suspected cholangiocarcinoma, EUS-guided liver biopsy and EUS FNA of pancreatic cystic neoplasm. Luminal interventions included gastroduodenal luminal biopsies and closure of perforated duodenal ulcer [47].

### Edge Comparative Studies

#### EDGE Vs Enteroscopy-Assisted ERCP

A multicenter study by Bukhari et al. published in 2018 compared the outcomes and adverse events between EUS-guided gastrogastrostomy-assisted ERCP (EUS-GG-ERCP) and enteroscopy-assisted ERCP (e-ERCP) in RYGB patients. Out of 60 patients, 30 underwent EUS-GG-ERCP and remaining 30 underwent e-ERCP (DBE in 19 and SBE in 11). Technical success was higher with EUS-GG-ERCP when compared to e-ERCP (100% vs 60%, *p* < .001). Total procedure time and median length of hospitalization was significantly shorter with EUS-GG ERCP group (49.9 min vs 90.7 min, p < .001; and 1 vs 10.5 days, *p* = .02). However, adverse event rate was similar in both the groups. (6.7% vs 10.0%, *p* = 1). No weight change was reported after EUS-GG-ERCP at mean follow up of 209 days [48•].

#### EDGE Vs LA-ERCP

A multicenter retrospective study published in 2018 by Kedia et al. compared the outcomes between EDGE and LA-ERCP. A total of 72 patients were included in the study (29 in EDGE group and 43 in LA-ERCP). Technical (96.5% vs 100%, *p* = 0.40) and clinical (96.5% vs 97.7%, *p* = 1.0) success rates were similar in the EDGE and LA-ERCP groups. In LA-ERCP, 21 patients had gastrostomy tube closure during the same session, whereas in 22 it was closed later. There was no significant difference in the adverse event rates between the groups (24% vs 19% *p* = 0.57). EDGE had significantly shorter procedure time and length of stay compared to LA-ERCP (73 min vs 184 min *p* < 0.00001; and 0.8 d vs 2.65 d *p* < 0.00008). The overall weight change after EDGE at mean follow up of 28 weeks was - 6.6 lbs. [49].

A meta-analysis presented by Khan et al. at DDW 2018 comparing LA-ERCP to EDGE included 22 observational studies (18 LA-ERCP and 4 EDGE) with 941 patients (843 LAERCP and 98 EDGE). Technical and clinical success rates were similar in both the groups (98% vs 96% *p* = 0.07 and 96% vs 96% *p* = 0.84) without any significant difference in the adverse event rate (13% vs 10%, *p* = 0.32). However, pooled mean length of stay and procedure time were shorter with EDGE (1.1 vs 3.1 days and 43 min vs 166 min) [50].

#### The Geisinger Experience

We presented our own experience from the Geisinger Medical Center comparing outcomes of EDGE vs LA-ERCP at The American College of Gastroenterology’s Annual Scientific Meeting, held in October 2019. A total of 76 RYGB patients who underwent ERCP (59 LA-ERCP and 17 EDGE) were analyzed. All cases of LA-ERCP and EDGE were performed in a single step setting. Technical and clinical success rates were 100% in both the groups. Adverse event rate and length of hospital stay were also similar (17% vs 6%, 2.7 vs 2.6 days, *p* = 0.94), however EDGE had significantly shorter procedure time when compared to LA-ERCP (103 min vs 208 min, *p* < 0.001). The median time for lumen-apposing metal stent removal was 22 days (range 0–111). There was no significant weight gain (−6.33 lbs.) at median follow up of 35 days in the EDGE group [51•].

**Fig. 7:**
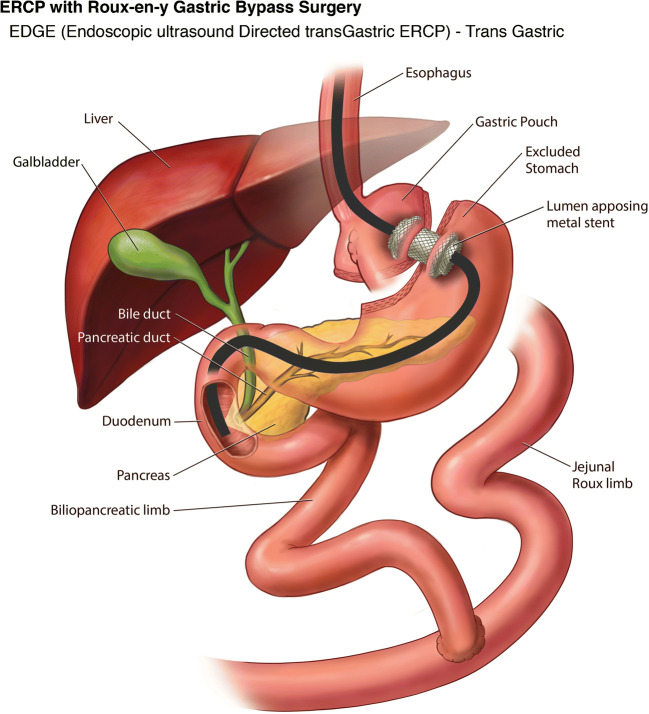
Endoscopic ultrasound-directed transgastric ERCP (EDGE) in Roux-en-Y Gastric Bypass: Transgastric access

**Fig. 8:**
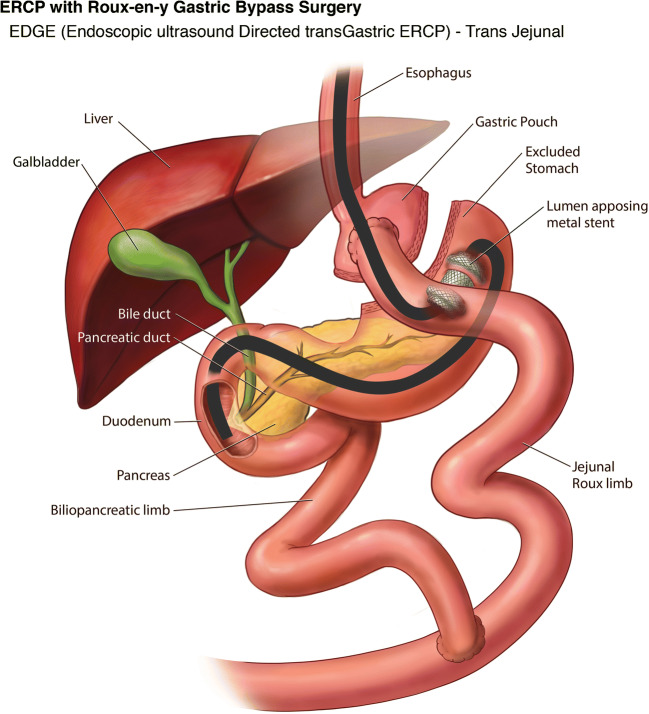
Endoscopic ultrasound-directed transgastric ERCP (EDGE) in Roux-en-Y Gastric Bypass: Transjejunal access

Based on our above experience, we now prefer to do LA-ERCP only when concomitant cholecystectomy needs to be performed. If RYGB patients are already post cholecystectomy and need an ERCP or access to excluded GI tract, the EDGE procedure is preferred. We perform all our EDGE cases as a single session procedure. Using a therapeutic linear echoendoscope, the excluded stomach is identified under endosonographic guidance looking for the “sand dollar sign” [52], preferentially as a gastro-gastric view when technically feasible, making sure that the distance between the two lumen is less than 10 mm. The excluded stomach is then punctured and injected with contrast under fluoroscopic guidance using a 19 g EUS-FNA needle. The excluded stomach is then distended using 250–400 ml of water mixed with indigo carmine solution via the EUS-FNA needle to create a safe target for LAMS placement. The EUS needle is then exchanged of, and the now available wider 20 mm electrocautery enhanced LAMS is placed freehand under EUS and fluoroscopic guidance to create a gastro-gastric access tract, without anchoring the stent. The LAMS lumen is then dilated using a through-the-scope balloon dilator to 20 mm after confirming reflux of the blue stained water. A diagnostic duodenoscope is then passed via the newly created gastro-gastrostomy for ampullary access or endoscopic intervention (Fig. 7). We have noticed almost no risk of stent migration with this technique. On occasion, we have created a jejuno-gastric access site using the 20 mm LAMS (Fig. 8); when a gastro-gastric access was not technically feasible either due to very small pouch size or lack of a safe gastro-gastric access window for the LAMS deployment. A follow up procedure is performed for LAMS removal usually within 2–3 weeks and the LAMS access site is actively closed using Endosuture with which no persistent fistula cases noted at our center. This more proactive approach for closure is partly influenced by our patients traveling long distances for their care and thereby we hope to reduce the need for reintervention or loss of follow up.

### Cost-Effectiveness

In this day and age of healthcare economics, decreasing the cost and length of stay are very important factors to be kept in mind. In a cost-analysis model comparing laparoscopic-assisted, enteroscopy-assisted, and EDGE-assisted ERCP approaches, EDGE was found to be more cost effective when compared to DAE-ERCP and LA-ERCP ($1431 vs $3147 and $9312) [53]. This was thought to be driven by the lack of need for the operating room and surgical supplies, and the associated costs. EDGE was also found to have the high total quality-adjusted life-years (QALY).

## Conclusions

In patients with altered Roux-en-Y anatomy, traditional ERCP using a duodenoscope is technically challenging and clinically ineffective for ampullary access. Over the decades multiple trials and techniques have been devised to reach the ampulla to perform therapeutic intervention for various pancreaticobiliary diseases. The combined techniques of using pediatric colonoscope and duodenoscope to even reach the ampulla have shown only 33%–67% success rate despite multiple attempts [12, 13]. With the development of DAE for small bowel evaluation and modifying its use to achieve ampullary access in RYGB anatomy, the biliary access rates improved to only 60%–65% [23••]. In 2002 when the LA-ERCP technique was developed, it surpassed the other advanced endoscopic techniques with higher technical and clinical success rates [25–27]. However, limitations remained as it is resource intensive, requiring collaboration of endoscopy and operating room schedules with the need for surgical access, and has higher complication rates and cost [30, 53]. To overcome these surgical limitations and to achieve an all-endoscopic alternative access, techniques such as PATENT were developed wherein gastrostomy tract was created endoscopically with the help of an enteroscope or EUS, without the need of surgical access. However, this still needed a percutaneous access which had its own set of complications from PEG site infections and need for PEG tract maturation prior to closure, delaying recovery and affecting quality of life [34–38].

In 2014, with the advent of LAMS, Kedia et al. first described an all-endoscopic “Internal EUS-Directed Transgastric ERCP (EDGE)” procedure and aptly termed it “Game Over”. This procedure has been called different names in different publications, such as EDGE, GATE, EDGI, EUS-TG-ERCP, and EUS-GG-ERCP. In essence, the procedure entails EUS-guided transgastric or transjejunal access into the remnant stomach followed by placement of a LAMS to facilitate the use of a standard duodenoscope for ERCP or other luminal or extraluminal endoscopic interventions.

This procedure has been a game changer in RYGB patients as it eliminates the need for surgical or percutaneous access, gives the option of repeat intervention for stent removal or exchange, and eliminates the need to keep a PEG tube in place for tract maturation, thereby reducing complication rates. All EDGE-related studies have demonstrated higher technical (100%) and clinical success rates (60%–100%) [41–47, 54]. EDGE has similar success rates (96.5% vs 100% *p* = 0.40) as compared to LA-ERCP in achieving therapeutic ERCP, but appears superior when compared to DAE (100% vs 60%, *p* < 0.001) [48–50]. Although LA-ERCP and EDGE have similar success rates, EDGE has shorter procedure time (73 vs 184 min, *p* < 0.00001) and post procedural hospital stay (0.8 vs 2.65 d, *p* < 0.00008) [49, 50].

In all the initial studies assessing the efficacy and safety of EDGE technique, a 15 mm diameter LAMS was used and a common problem encountered during the procedure was stent dislodgement (15.4%–20%) [42, 43]. In addition to stent dislodgement, other adverse events such as bleeding (7.6%), LAMS malposition (4.5%), migration (4.5%), perforation (1.5%) and pancreatitis (1.5%) were also reported [55]. Some studies have noticed that the gastrogastric access had a higher complication rate than jejunogastric, but stent dislodgment was higher with the jejunogastric access.

To decrease the risk of stent dislodgment, EDGE was performed in two-sessions by the endoscopists with a mean wait time of 11–48 days to allow fistula tract maturation between LAMS placement and ERCP [42, 44]. To date, there is one prior report where the procedure was delayed only 72 h rather than a wait of 11–48 days when larger size (20 mm) LAMS was used [56]. Another study at DDW 2019 demonstrated the use of 20 mm LAMS. A total of nine patients were included in the study; technical and clinical success was 100%. The mean wait time was 2 days between the stent placement and the ERCP without any reported stent dislodgment [54]. In addition to the size of LAMS, no stent dislodgement was noticed when slim duodenoscope was used instead of therapeutic duodenoscope [42]. This evidence was supported with the research study at our own institution where stent dislodgement occurred only while using a therapeutic duodenoscope in a single case, and we have been able to perform all our EDGE cases as a same session ERCP without any wait time after LAMS deployment with the combination of gastrogastric access with 20 mm LAMS and use of the slim duodenoscope, making it a true immediate access single session fully endoscopic procedure [51•].

The outcomes of LAMS site fistula closure are also of concern as it may be associated with the risk of weight regain in these high-risk patients. No significant weight changes were reported from the time of LAMS insertions to removal and fistula closure while LAMS was left in place after ERCP for an average of 20–82 days [41–46]. Many modalities like APC, OTSC, endoscopic suturing, and double pigtail plastic stent have been described to close the EDGE access tract after ERCP. Double pigtail plastic stent was considered more advantageous compared to other techniques in terms of its cost, endoscopic approach and minimal technical support in one recent study [46•] (Table 1).

**Table 1:** Summary of all EDGE papers reported to date

Study Publication	Type of LAMS: Cautery enhanced (CE), Non cautery enhanced (NCE)	Size of LAMS	Type of Fistula: Gastrogastric (GG), Jejunogastric (JG)	Technical success	Clinical success	No. of sessions	LAMS removal median/mean time	Type of closure: Endoscopic suturing (ES), Over the scope clip (OTSC), Argon plasma coagulation (APC), Through the scope clip (TTS)	Persistent fistula	Adverse events	Weight changes
Kedia et al. 2015 (*n* = 5)	NR^a^	15 mm	GG = 4 JG = 1	100%	60%	one session = 3; two session = 2	3 weeks	ES = 2	none	stent dislodgement (60%)	none
Tyberg et al. 2016 (*n* = 16)	NR	15 mm	GG = 6 JG = 10	100%	91%	one session = 4; two sessio*n* = 6	NR	ES = 7, OTSC = 2 secondary intention =1	1 out of 8	stent dislodgment (19%), jejunal perforation in one patient	negative 2.85 kg
Ngamruengphong et al. 2017 (*n* = 13)	CE = 5 NCE = 8	15 mm	GG = 6 JG = 7	100%	100%	one session =2; two session = 11	20 days	ES = 6, OTSC = 3, APC = 3	1 out of 12	stent dislodgment (33%)	loss of 3.6± 4.8 kg
James et al. 2018 (*n* = 19)	CE = 14, NCE = 5	15 mm	GG = 8 JG = 11	100%	100%	one session =4; two session = 15	182 days	APC = 12 None = 5	1 out of 11	stent malposition (6/19)	weight gain of 1.7 kg
Bukhari et al. 2018 (*n* = 30)	CE = 14 NCE = 16	15 mm	GG =17 JG =13	100%	100%	one session =8; two session =22	26 days	ES = 8 OTSC = 7 APC = 15	1 of 30	LAMS migration (6.7%), bleeding (3.3%)	neg 1.1± 6.1 kg
Chiang et al. 2018 (*n* = 66)	CE = 33	NR	GG = 30 JG = 34	92.40%	NR	one session = 43	NR	NR	NR	bleeding (7.6%), LAMS malposition (4.5%), LAMS migration (4.5%), perforation (1.5%), pancreatitis (1.5%)	NR
Kedia et al. 2018 (*n* = 29)	NR	15 mm	GG = 28	96.50%	96.50%	NR	NR	NR	NR	perforation (1), pancreatitis (2) stent dislodgement (3) bleeding (1)	negative 6.6 lbs
Wang et al. 2019 (*n* = 10)	NR	15 mm	GG = 3 JG = 7	100%	100%	one session =7; two session =2	14–217 days	exchanged with Plastic double pigtail stent	None	stent dislodgment (20%), bleeding in one patient	NR
Hsueh et al. 2019 (*n* = 9)	NR	20 mm	GG = 5 JG = 4	100%	100%	one session =2; two session =7	NR	NR	NR	none	NR
Runge et al. 2019 (*n* = 166)	NR	NR	GG = 86 JG = 80	98%	NR	one sessio*n* = 85; two session = 81	47 days	ES =51 APC = 46 OTSC = 15 TTS = 6 None = 44	10 of 85	perforation (4.2%), stent migration (0.6%), bleeding, pneumoperitoneum (1.3%), post ERCP pancreatitis (2%), sepsis (0.6%), GGF leakage (0.6%).	NR
Kraft et al. 2019 (*n* = 14)	CE = 12 NCE = 2	20 mm (n = 8) 15 mm (n = 6)	GG = 8 JG = 6	100%	100%	One session = 5; two session = 9	38 days	Spontaneous closure = 7, APC = 3	NONE	Stent dislodgement (14.3%)	NR

^a^*NR* = Not Reported

Our review has shown strong data in support of EDGE as an all-endoscopic, efficacious, safer and superior alternative in terms of cost and time and that can be performed as a single-session procedure using minimal resources. However, LA-ERCP can be considered in patients who need simultaneous cholecystectomy. In order for EDGE to evolve into a more effective standardized procedure across the board, prospective randomized studies are needed to compare the size of LAMS used, type of duodenoscope used, one vs two session procedure to allow for tract maturity, need for anchoring the stent with endosuture or OTSC, assessing its value for ERCP vs other endoscopic interventions in the bypassed GI tract, and comparing the modalities of fistula closure vs spontaneous closure with respective response. Dedicated procedure billing codes are also needed to better code and bill for this procedure, taking into consideration all the morbidity benefits, patient convenience, and cost savings as compared to surgical alternatives.

A systematic approach is necessary in managing these patients with pancreaticobiliary disease with underlying RYGB anatomy, with close collaborations between GI, radiology, interventional radiology and surgery. A multidisciplinary approach is key in deciding the most optimal method in managing these patients based on available expertise, resources, surgical and radiological back up, experience of the endoscopist and the staff in handling these patients and the associated complications, as well as patient’s comorbidities and preference. Further prospective studies will help guide, standardize practices and management approaches for this population.

## Abbreviations

RYGB: Roux-en-Y Gastric Bypass
ERCP: Endoscopic Retrograde Cholangiopancreatography
EDGE: Endoscopic ultrasound Directed transGastric ERCP
DAE: Device Assisted Enteroscopy
DBE: Double Balloon Enteroscopy
SBE: Single Balloon Enteroscopy
SE: Spiral Enteroscopy
LA-ERCP: Laparoscope-Assisted ERCP
PATENT: Percutaneous Assisted Transprosthetic Endoscopic Therapy
PEG: Percutaneous Endoscopic Gastrostomy
FCSEMS: Full Covered Self-Expandable Metal Stent
SEMS: Self- Expandable Metal Stent
EUS: Endoscopic Ultrasonography
LAMS: Lumen Apposing Metal Stent
EUS-TG-ERCP: EUS-guided TransGastric ERCP
OTSC: Over The Scope Clip
APC: Argon Plasma Coagulation
GATE: Gastric Access Temporary for Endoscopy
EDGI: EUS-Directed transGastric Intervention
ESD: Endoscopic Submucosal Dissection
DDW: Digestive Disease Week
GGF: Gastro-Gastric Fistula
EUS-GG-ERCP: EUS-guided GastroGastrostomy-assisted ERCP
e-ERCP: Enteroscopy-assisted ERCP
FNA: Fine needle aspiration

## References

[1] Mokdad AH, Marks JS, Stroup DF, Gerberding JL (2004). Actual causes of death in the United States, 2000. JAMA.

[2] Solomon CG, Dluhy RG (2004). Bariatric surgery — quick fix or long-term solution?. N Engl J Med.

[3] Sjöström L, Lindroos A-K, Peltonen M, Torgerson J, Bouchard C, Carlsson B, Dahlgren S, Larsson B, Narbro K, Sjöström CD, Sullivan M, Wedel H (2004). Lifestyle, diabetes, and cardiovascular risk factors 10 years after bariatric surgery. N Engl J Med.

[4] Schauer PR, Ikramuddin S (2001). Laparoscopic surgery for morbid obesity. Surg Clin North Am.

[5] Santry HP, Gillen DL, Lauderdale DS (2005). Trends in bariatric surgical procedures. J Am Med Assoc.

[6] Shiffman ML, Sugerman HJ, Kellum JH, Brewer WH, Moore EW (1993). Gallstones in patients with morbid obesity. Relationship to body weight, weight loss and gallbladder bile cholesterol solubility. Int J Obes Relat Metab Disord.

[7] Nagem RG, Lázaro-da-Silva A, de Oliveira RM, Morato VG (2012). Gallstone-related complications after roux-en-Y gastric bypass: a prospective study. Hepatobiliary Pancreat Dis Int.

[8] Lopes TL, Wilcox CM (2010). Endoscopic retrograde Cholangiopancreatography in patients with roux-en-Y anatomy. Gastroenterol Clin N Am.

[9] Lee S, Sahagian KG, Schriver JP (2006). Relationship between varying roux limb lengths and weight loss in gastric bypass. Curr Surg.

[10] Gostout CJ, Bender CE (1988). Cholangiopancreatography, sphincterotomy, and common duct stone removal via roux-en-Y limb enteroscopy. Gastroenterology.

[11] Elton E, Hanson BL, Qaseem T, Howell DA (1998). Diagnostic and therapeutic ERCP using an enteroscope and a pediatric colonoscope in long-limb surgical bypass patients. Gastrointest Endosc.

[12] Hintze RE, Adler A, Veltzke W, Abou-Rebyeh H (1997). Endoscopic access to the papilla of Vater for endoscopic retrograde Cholangiopancreatography in patients with Billroth II or roux-en-Y Gastrojejunostomy. Endoscopy.

[13] Wright BE, Cass OW, Freeman ML (2002). ERCP in patients with long-limb roux-en-Y gastrojejunostomy and intact papilla. Gastrointest Endosc.

[14] Wang TJ, Ryou M (2018). Evolving techniques for endoscopic retrograde cholangiopancreatography in gastric bypass patients. Curr Opin Gastroenterol.

[15] Yamamoto H, Sekine Y, Sato Y, Higashizawa T, Miyata T, Iino S, Ido K, Sugano K (2001). Total enteroscopy with a nonsurgical steerable double-balloon method. Gastrointest Endosc.

[16] Aabakken L, Bretthauer M, Line P (2007). Double-balloon enteroscopy for endoscopic retrograde cholangiography in patients with a roux-en-Y anastomosis. Endoscopy.

[17] Dellon ES, Kohn GP, Morgan DR, Grimm IS (2009). Endoscopic retrograde Cholangiopancreatography with single-balloon Enteroscopy is feasible in patients with a prior roux-en-Y anastomosis. Dig Dis Sci.

[18] Skinner M, Popa D, Neumann H, Wilcox C, Mönkemüller K (2014). ERCP with the overtube-assisted enteroscopy technique: a systematic review. Endoscopy.

[19] De Koning M, Moreels TG (2016). Comparison of double-balloon and single-balloon enteroscope for therapeutic endoscopic retrograde cholangiography after roux-en-Y small bowel surgery. BMC Gastroenterol.

[20] Akerman PA, Cantero D (2009). Spiral Enteroscopy and push Enteroscopy. Gastrointest Endosc Clin N Am.

[21] Ali MF, Modayil R, Gurram KC, Brathwaite CEM, Friedel D, Stavropoulos SN (2018). Spiral enteroscopy-assisted ERCP in bariatric-length roux-en-Y anatomy: a large single-center series and review of the literature (with video). Gastrointest Endosc.

[22] El Zouhairi M, Watson JB, Desai SV, Swartz DK, Castillo-Roth A, Haque M (2015). Rotational assisted endoscopic retrograde cholangiopancreatography in patients with reconstructive gastrointestinal surgical anatomy. World J Gastrointest Endosc.

[23.••] Shah RJ, Smolkin M, Yen R, Ross A, Kozarek RA, Howell DA, et al. A multicenter, U.S. experience of single-balloon, double-balloon, and rotational overtube–assisted enteroscopy ERCP in patients with surgically altered pancreaticobiliary anatomy (with video). Gastrointest Endosc 2013:77:593–600. doi:10.1016/j.gie.2012-10-015. **This is the only multicenter study comparing the technical and clinical success rates of all the three type of enteroscopy assisted techniques**. 10.1016/j.gie.2012.10.015 23290720

[24] Peters M, Papasavas PK, Caushaj PF, Kania RJ, Gagné DJ (2002). Laparoscopic transgastric endoscopic retrograde cholangiopancreatography for benign common bile duct stricture after roux-en-Y gastric bypass. Surg Endosc.

[25] Banerjee N, Parepally M, Byrne TK, Pullatt RC, Coté GA, Elmunzer BJ (2017). Systematic review of transgastric ERCP in roux-en-Y gastric bypass patients. Surg Obes Relat Dis.

[26.•] Abbas AM, Strong AT, Diehl DL, Brauer BC, Lee IH, Burbridge R, et al. Multicenter evaluation of the clinical utility of laparoscopy-assisted ERCP in patients with Roux-en-Y gastric bypass. Gastrointest Endosc 2018:87:1031–9. Doi:10.1016/j.gie.2017-10-044. **This article summarized the results pooled from multiple centers thus providing with ‘p-value’ focussing on technical and clinical success rates, time taken to perform the procedure, and adverse events of laparoscopy-assissted ERCP technique.** 10.1016/j.gie.2017.10.044 29129525

[27] Saleem A, Levy MJ, Petersen BT, Que FG, Baron TH (2012). Laparoscopic assisted ERCP in roux-en-Y gastric bypass (RYGB) surgery patients. J Gastrointest Surg.

[28] Desai SV, Naveed M, Jazwinski A, Jowell PS, Branch MS (2011). 299 spiral Enteroscopy versus laparoscopic-assisted endoscopy for completion of ERCP in patients with roux-en-Y gastric bypass surgery. Gastrointest Endosc.

[29] da Ponte-Neto AM, Bernardo WM, de A. Coutinho LM, Josino IR, Brunaldi VO, Moura DTH, et al. (2018). Comparison between Enteroscopy-based and laparoscopy-assisted ERCP for accessing the biliary tree in patients with roux-en-Y gastric bypass: systematic review and meta-analysis. Obes Surg.

[30] Choi EK, Chiorean MV, Coté GA, El Hajj II, El Hajj I, Ballard D (2013). ERCP via gastrostomy vs. double balloon enteroscopy in patients with prior bariatric roux-en-Y gastric bypass surgery. Surg Endosc.

[31] Snauwaert C, Laukens P, Dillemans B, Himpens J, De Looze D, Deprez PH (2015). Laparoscopy-assisted transgastric endoscopic retrograde cholangiopancreatography in bariatric roux-en-Y gastric bypass patients. Endosc Int Open.

[32] Paranandi B, Joshi D, Mohammadi B, Jenkinson A, Adamo M, Read S, Johnson GJ, Chapman MH, Pereira SP, Webster GJ (2016). Laparoscopy-assisted ERCP (LA-ERCP) following bariatric gastric bypass surgery: initial experience of a single UK centre. Frontline Gastroenterol.

[33] Shuster D, Elmunzer BJ (2014). What is the preferred approach to performing endoscopic retrograde cholangiopancreatography in patients with roux-en-Y gastric bypass anatomy?. Gastroenterology.

[34] Baron TH, Wong Kee Song LM (2012). Percutaneous assisted transprosthetic endoscopic therapy (PATENT): expanding gut access to infinity and beyond! (with video). Gastrointest Endosc.

[35.••] Baron TH, Song LMWK, Ferreira LEVV, Smyrk TC. Novel approach to therapeutic ERCP after long-limb Roux-en-Y gastric bypass surgery using transgastric self-expandable metal stents: experimental outcomes and first human case study (with videos). Gastrointest Endosc 2012:75:1258–63. Doi:10.1016/j.gie.2012-02-026. **This review first demonstrated the PATENT technique in animals and humans. In this technique ERCP was performed via the PEG tube by the endoscopist without the need of surgical assisstance unlike in LAERCP.** 10.1016/j.gie.2012.02.026 22624815

[36] Law R, Wong Kee Song L, Petersen B, Baron T (2013). Single-session ERCP in patients with previous roux-en-Y gastric bypass using percutaneous-assisted transprosthetic endoscopic therapy: a case series. Endoscopy.

[37] Law R, Grimm I, Baron T (2016). Modified percutaneous assisted transprosthetic endoscopic therapy for transgastric ERCP in a gastric bypass patient. Endoscopy.

[38] Attam R, Leslie D, Freeman M, Ikramuddin S, Andrade R (2011). EUS-assisted, fluoroscopically guided gastrostomy tube placement in patients with roux-en-Y gastric bypass: a novel technique for access to the gastric remnant. Gastrointest Endosc.

[39] Kedia P, Kumta N, Widmer J, Sundararajan S, Cerefice M, Gaidhane M, Sharaiha R, Kahaleh M (2015). Endoscopic ultrasound-directed transgastric ERCP (EDGE) for roux-en-Y anatomy: a novel technique. Endoscopy.

[40.••] Kedia P, Sharaiha RZ, Kumta NA, Kahaleh M. Internal EUS-directed transgastric ERCP (EDGE): game over. Gastroenterology 2014:147:566–8. Doi:10.1053/j.gastro.2014-05-045. **This is the first reported publication describing the use of LAMS to create a completely endoscopic access approach for ERCP in RYGB patients, and coined the term “EDGE” for this procedure.** 10.1053/j.gastro.2014.05.045 24975458

[41.•] Kedia P, Tyberg A, Kumta NA, Gaidhane M, Karia K, Sharaiha RZ, et al. EUS-directed transgastric ERCP for roux-en-Y gastric bypass anatomy: a minimally invasive approach. Gastrointest Endosc 2015:82:560–5. Doi:10.1016/j.gie.2015-03-1913. **One of the earliest studies on the EDGE technique by the initial authors describing the technical and clinical success rates in the first case series.** 10.1016/j.gie.2015.03.1913 25952086

[42] Ngamruengphong S, Nieto J, Kunda R, Kumbhari V, Chen Y-I, Bukhari M, el Zein M, Bueno R, Hajiyeva G, Ismail A, Chavez Y, Khashab M (2017). Endoscopic ultrasound-guided creation of a transgastric fistula for the management of hepatobiliary disease in patients with roux-en-Y gastric bypass. Endoscopy.

[43] Tyberg A, Nieto J, Salgado S, Weaver K, Kedia P, Sharaiha RZ, Gaidhane M, Kahaleh M (2017). Endoscopic ultrasound (EUS)-directed Transgastric endoscopic retrograde Cholangiopancreatography or EUS: mid-term analysis of an emerging procedure. Clin Endosc.

[44.•] James TW, Baron TH. Endoscopic Ultrasound-Directed Transgastric ERCP (EDGE): a Single-Center US Experience with Follow-up Data on Fistula Closure. Obes Surg 2019:29:451–6. Doi:10.1007/s11695-018-3531-2. **In addition to success rates, this article shared the experience of fistula status and its effects on weight changes following the EDGE technique which has thrown more light into the details of the minor complications and helped the other authors to aviod them.** 10.1007/s11695-018-3531-2 PMC6550479 30302653

[45] Runge TM, Kowalski TE, Baron TH, Chiang AL, James T, Schlachterman A, Loren DE, Nieto J, Khara HS, Diehl DL, Confer B, Kumar SV, Irani SS, Nasr J, Krafft MR, Hsueh W, Law R, Patel AH, Stevens T, Chahal P, al-Haddad MA, Pleskow DK, Faisal MF, Huggett MT, Trindade A, Ichkhanian Y, Vosoughi K, Brewer Gutierrez OI, Yang J, Dbouk M, Ngamruengphong S, Kumbhari V, Singh V, Kalloo AN, Khashab MA (2019). 1026 living on the the EDGE- success, long-term complications, and implications following EUS-directed Transgastric ERCP:A multicenter study. Gastrointest Endosc.

[46.•] Wang TJ, Thompson CC, Ryou M. Gastric access temporary for endoscopy (GATE): a proposed algorithm for EUS-directed transgastric ERCP in gastric bypass patients. Surg Endosc 2019:33:2024–33. Doi:10.1007/s00464-019-06715-z. **This article coined an alternative term for EDGE, called GATE technique, and in addition, provided insight on new fistula closure techniques and outcomes.** 10.1007/s00464-019-06715-z 30805786

[47] Krafft MR, Hsueh W, James TW, Runge TM, Baron TH, Khashab MA, Irani SS, Nasr JY (2019). The EDGI new take on EDGE: EUS-directed transgastric intervention (EDGI), other than ERCP, for roux-en-Y gastric bypass anatomy: a multicenter study. Endosc Int Open.

[48.•] Bukhari M, Kowalski T, Nieto J, Kunda R, Ahuja NK, Irani S, Shah A, Loren D, Brewer O, Sanaei O, Chen YI, Ngamruengphong S, Kumbhari V, Singh V, Aridi HD, Khashab MA An international, multicenter, comparative trial of EUS-guided gastrogastrostomy-assisted ERCP versus enteroscopy-assisted ERCP in patients with roux-en-Y gastric bypass anatomy. Gastrointest Endosc 2018:88:486–94. Doi:10.1016/j.gie.2018-04-2356. 10.1016/j.gie.2018.04.2356 29730228

[49] Kedia P, Tarnasky PR, Nieto J, Steele SL, Siddiqui A, Xu M-M, Tyberg A, Gaidhane M, Kahaleh M (2019). EUS-directed Transgastric ERCP (EDGE) versus laparoscopy-assisted ERCP (LA-ERCP) for roux-en-Y gastric bypass (RYGB) anatomy: A multicenter early comparative experience of clinical outcomes. J Clin Gastroenterol.

[50] Khan MA, Kedia P, Tyberg A, Shrestha S, Ismail MK, Gaidhane M, Tarnasky PR, Kahaleh M (2018). Mo1338 comparison of EUS directed Transgastric endoscopic retrograde Cholangiopancreatography in patients with roux-en-Y bypass: A meta-analysis. Gastrointest Endosc.

[51.•] Parvataneni S, Kumar V, Confer B, Diehl DL, Khara HS. EUS-Directed Transgastric ERCP (EDGE) versus Laparoscopy-Assisted ERCP (LAERCP) for Roux-en-Y Gastric Bypass (RYGB): A Single Center Experience. Am J Gastroenterol 2019:114:S536. Doi:10.14309/01.ajg.0000593204-85987-e7. **This article demonstrates our own experiences and outcomes with the EDGE technique as compared to LAERCP, demonstrating the use of the newer larger diameter 20 mm LAMS for EDGE.**

[52] Siddiki H, Baron TH (2018). The sand dollar sign: a reliable EUS image to identify the excluded stomach during EUS-guided gastrogastrostomy. Gastrointest Endosc.

[53] James HJ, James TW, Wheeler SB, Spencer JC, Baron TH (2019). Cost-effectiveness of endoscopic ultrasound-directed transgastric ERCP compared with device-assisted and laparoscopic-assisted ERCP in patients with roux-en-Y anatomy. Endoscopy.

[54] Hsueh W, Krafft MR, Abdelqader A, Nasr J (2019). Su1167 EUS-directed Transgastric ERCP with 20 MM lumen-apposing metal stents in patients with roux-en-Y gastric bypass, are we closer to perfection?. Gastrointest Endosc.

[55] Chiang AL, Gaidhane M, Loren DE, Kahaleh M, Schlachterman A, Millman J, Tyberg A, Nieto J, Kedia P, Tarnasky PR, Raijman I, Khara HS, Diehl DL, Prabhu A, Kowalski TE (2018). 338 impact of EUS-directed Transgastric ERCP (EDGE procedure) access route on technical success and adverse events: A multicenter experience. Gastrointest Endosc.

[56] Vallabh H, Poushanchi B, Hsueh W, Tabone L, Nasr J (2018). EUS-directed transgastric ERCP (EDGE) with use of a 20-mm × 10-mm lumen-apposing metal stent in a patient with roux-en-Y gastric bypass. VideoGIE.

